# *Drosophila* Ste-20 Family Protein Kinase, Hippo, Modulates Fat Cell Proliferation

**DOI:** 10.1371/journal.pone.0061740

**Published:** 2013-04-18

**Authors:** Hongling Huang, Wenqing Wu, Lei Zhang, Xin-Yuan Liu

**Affiliations:** 1 State Key Laboratory of Cell Biology, Institute of Biochemistry and Cell Biology, Shanghai Institutes for Biological Sciences, Chinese Academy of Sciences, Shanghai, P.R. China; 2 Xinyuan Institute of Medicine and Biotechnology, College of Biological Sciences, Zhejiang Sci-Tech University, Hangzhou, China; Simon Fraser University, Canada

## Abstract

**Background:**

Evolutionarily conserved Hippo (Hpo) pathway plays a pivotal role in the control of organ size. Although the Hpo pathway regulates proliferation of a variety of epidermal cells, its function in non-ectoderm-derived cells is largely unknown.

**Methodology/Principal Findings:**

Through methods including fat quantification assays, starvation assays, *in vivo* labeling assays, we show that overexpression of Hpo in *Drosophila melanogaster* fat body restricts *Drosophila* body growth and reduces fat storage through regulation of adipocyte proliferation rather than through influencing the size of fat cells and lipid metabolism, whereas compromising Hpo activity results in weight gain and greater fat storage. Furthermore, we provide evidence that Yorkie (Yki, a transcriptional coactivator that functions in the Hpo pathway) antagonizes Hpo to modulate fat storage in *Drosophila*.

**Conclusions/Significance:**

Our findings specify a role of Hpo in controlling mesoderm-derived cell proliferation. The observed anti-obesity effects of Hpo may indicate great potential for its utilization in anti-obesity therapeutics.

## Introduction

The evolutionarily conserved Hippo (Hpo) signaling pathway has emerged as a pivotal pathway in the control of organ size. Since the discovery of serine/threonine kinase Warts (Wts), the first member of the Hpo pathway [1], [2], extensive studies have been done to search for novel components of this pathway, resulting in the uncovering of the core members of Hpo pathway. In general, the Hpo pathway can be divided into three parts: the upstream regulators, the core kinase cassette, and downstream transcriptional effectors [3]. The core kinase cassette of the Hpo pathway, which consists of the serine/threonine Ste20-like kinase Hpo [4], [5], the nuclear Dbf-2-related (NDR) family kinase Wts, and their scaffold proteins Salvador (Sav) [6] and Mob as tumor suppressor (Mats) [7], acts through sequential phosphorylation events to sequester the growth-promoting transcriptional coactivator Yorkie (Yki) [8] in the cytoplasm. Without suppression from the Hpo signaling pathway, Yki enters nucleus and associates with transcription factors, such as Scalloped (Sd) [9], [10], Homothorax, Teashirt [11], and Mad [12] to induce the expression of such target genes, as bantam, cyclinE, and diap1, thereby promoting proliferation and inhibiting apoptosis.

Recently, Echinoid [13], Pez [14], *Drosophila* Homeodomain-interacting protein kinases [15] and vertebrate non-receptor tyrosine phosphatase 14 (PTPN14) [16] have been reported as new modulators of the Hpo pathway. Other studies have been performed to determine various functions of the pathway. It has been reported that the Hpo pathway regulates tissue growth by balancing cell proliferation and apoptosis and also participates in stem cell maintenance, tissue homeostasis and regeneration [17]. Dysfunction of the Hpo pathway in *Drosophila* wing and eye epithelial cells leads to overgrown wings and compound eyes, while its dysfunction in vertebrate epithelial cells causes a wide variety of tumors [18]. Although the critical role of the Hpo pathway in regulating epidermal cells has been well established, whether the pathway also controls non-ectoderm-derived cells is elusive. Interestingly, it has been reported that the Hpo pathway can restrain cardiomyocyte proliferation and heart size through inhibition of Wnt signaling [19], indicating that the Hippo pathway may control the proliferation of cells derived from different cell layers.

Obesity has become an increasingly prevalent problem worldwide [20]. It may result in a variety of complications, such as insulin resistance, type 2 diabetes, cardiovascular disease, hypertension, and certain cancers [21], [22]. Although several strategies for prevention and treatment of obesity have been proposed, including diet control, exercise, drug therapy, bariatric surgery, or a combination of these strategies [23], fast and effective anti-obesity strategy remains largely elusive. Obesity results from imbalance in caloric intake and expenditure, and is characterized as an increase in adipocyte size or number [24], [25], [26]. Several clinical drugs have been designed to combat obesity through the restriction of fat absorption from gut (such as Orlistat and Cetilistat) and the promotion of fat-burning (such as AOD9604) [27]. Additional research targeting fat metabolism, including adipogenesis, has also been carried out [28]. Furthermore, control of fat cell number, either by adipocyte deletion through apoptosis or by restricting fat cell proliferation, has emerged as new strategy for obesity treatment [29].

*Drosophila melanogaster* has been well established as a relevant model system to investigate and understand many human diseases, including Parkinson's disease [30], Huntington's disease [31], Alzheimer's disease [32]. Several studies strongly suggest that *Drosophila* can also act as a model for investigating fat storage. The *Drosophila* adipose tissue, or fat body, that is responsible for storing energy [33] is derived from the mesoderm [34]. Further, the evolutionarily conserved insulin pathway plays a similar role in the regulation of fat content in *Drosophila* and vertebrates [35]. In addition, brummer lipase is an evolutionarily conserved fat storage regulator involves in the conversion of triglycerides to fatty acids [36], indicating that fat composition and mechanisms of lipid storage and utilization are also evolutionarily conserved [33]. Several other reports suggested that the regulations of adipogenesis by some genes/pathways are also conserved, such as Adipose (Adp) [37] and Hedgehog [38] signaling.

In this study, we use *Drosophila* as a model system to investigate the mechanism of fat cell proliferation. Our *in vivo* evidence indicated that the gain of function of Hpo in *Drosophila* fat body results in reduction of fat storage, while depletion of Hpo produces obese flies; therefore, we conclude that Hpo acts as a regulator of fat cell proliferation.

## Materials and Methods

### Fly stocks

The following fly stocks were used in this study: *UAS-Flag-Hpo* [39], *UAS-Hpo-RNAi* (V104169, VDRC), *UAS-myc-Yki(S168A)* [10], *hs-flp act>CD2>Gal4* [*10*], *Dcg-Gal4*; *Dcg-GFP* (a gift from Graff, J.M., University of Texas Southwestern Medical Center). The *Hpo^BF33^* null allele was described previously [40]. All of the *Drosophila* were cultured at 25°C.

### Body Weight Measurement, Triglyceride quantification, Nile Red staining and Oil Red O staining, and Fat Body Visualization

For body weight measurement, third instar larvae or five to seven-day-old flies were weighed in batches of more than ten. Data were expressed as mean ±SEM from at least three independent experiments.

In order to determinate triglyceride content, third instar larvae or five to seven-day-old flies were homogenized in a 1∶2 methanol∶chloroform mixture and then separated by adding distilled water to generate a lower chloroform layer and an upper aqueous methanol layer. The crude lipid extracted from chloroform layer was air-dried and re-dissolved in 1% Triton-X/Ethanol. To quantify the triglyceride level, the Infinity Triglyceride Reagent (F6428 and T2449, Sigma-Aldrich) was used according to the manufacturer's instructions. The triglyceride content was normalized by the weight of larvae or adult fly.

For Nile Red staining, the fat body from third instar larvae was dissected, fixed in formalin, permeabilized in 0.1% Triton X-100/PBS and then stained with Nile Red solution (72485, Sigma-Aldrich). The sample was mounted and scanned by confocal laser scanning microscopy (SP5, Leica microsystems).

For Oil Red O staining, third instar larvae were dissected in PBS to expose the fat bodies. After fixation in buffered formalin, samples were incubated in freshly prepared Oil Red O solution (four parts water mixed with six parts 0.5% Oil Red O in isopropanol) for 30 min and then were washed several times with distilled water and once with 60% isopropanol to remove excess stain. The remaining Oil Red O was extracted from stained cells with 100% isopropanol, and the absorbance at 492 nm was measured to quantify the dye.

To visualize fat bodies, intact third instar larvae were asphyxiated in methanol prior to microscopic analysis. The image was taken by fluorescence stereomicroscope (SZX16, Olympus).

### Generation of mosaic analysis with a repressible cell marker (MARCM) clone and flip-out clone

Mutant clones in the fat body were generated by the MARCM system as reported in the literature [41], [42]. Briefly, embryos whose cells have not yet undergone fat cell fate determination were collected 3–6 hours after egg deposition and then subjected to heat shock for 1 h at 37°C and allowed to develop to third instar larvae at 25°C. The genotypes for generating *hpo* null clones and control clones are as follows: *hsflp*, *UAS-GFP*; *FRT42D ubi-Gal80/FRT42DHpo^BF33^ and hsflp*, *UAS-GFP*; *FRT42Dubi-Gal80/FRT42D*. To generate flip-out clones, *UAS-Hpo* transgenic flies were crossed with *hsflp*, *act>CD2>Gal4* flies and the clones were induced at 72–96 hours after egg deposition (AED) by heat-shock for 20–30 minutes at 37°C.

### Starvation Assay

The starvation experiments were carried out with five to seven-day-old male flies. Newly enclosed flies representing each genotype were raised under identical conditions in standard cornmeal medium until the beginning of the starvation treatment. To starve the flies, batches of 15–25 flies of each genotype were transferred to vials containing 2% agarose. Dead flies were counted every 8–12 hours for survival rate calculations. Average survival rate of 3–5 vials of each genotype was measured.

### Preparation of DNA from fat body

The fat body was dissected and then lysed in lysis buffer (200 mM Tris-Cl, pH 7.5, 30 mM EDTA, 2% SDS, 200 µg/ml Proteinase K) for over three hours. After removing RNA and protein through the addition of RNaseA and protein precipitation buffer, the genomic DNA was precipitated by isopropanol and then dissolved in distilled water. The concentration of the resulting DNA sample was determined by measuring the absorbance at 260 nm.

### Statistical Analysis

All data were expressed as mean ± SEM and were analyzed using Student's t-test by R 2.9.0. Results were considered statistically significant when p<0.05.

## Results

### Overexpression of Hpo in fat body reduces overall growth and fat storage of *Drosophila*

**Figure 1:**
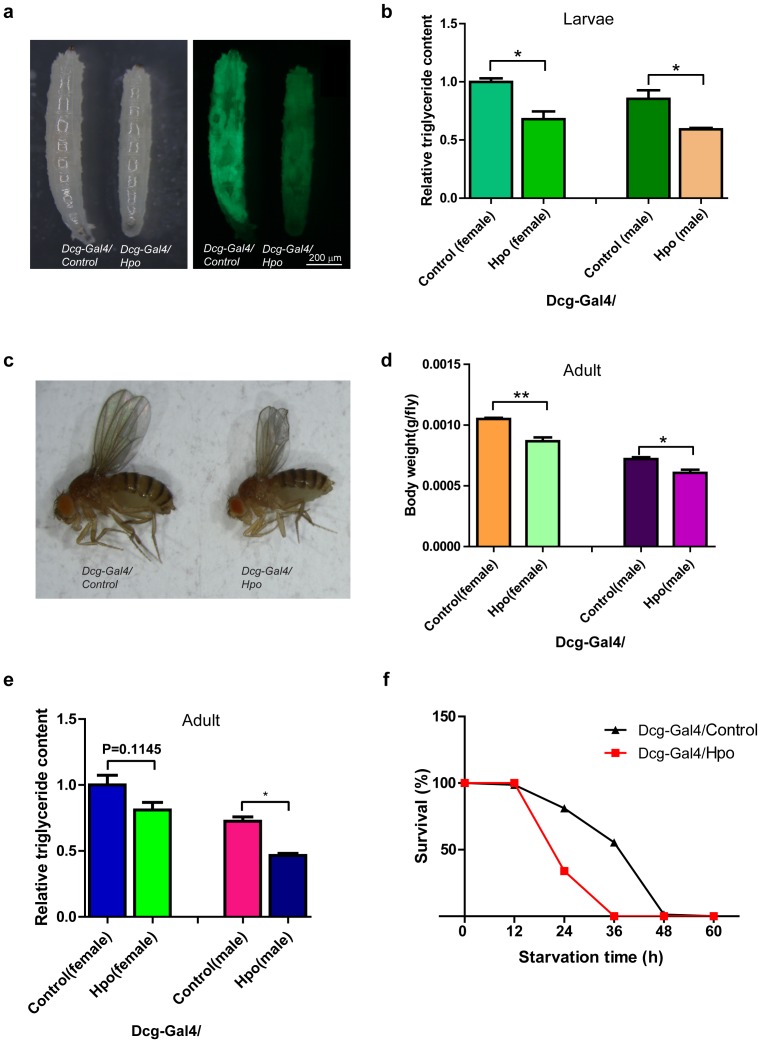
Activating Hpo inhibits fat accumulation in *Drosophila melanogaster*. (a) Ectopic Hpo reduces larval body size and fat body reporter signal. Larvae carrying a fat body GFP reporter (Dcg-GFP) expressing either control or Hpo transgene driven by *Dcg-Gal4* in fat body were photographed under bright field (left) or GFP fluorescence (right) microscopy. Note that body size and GFP fluorescence signal were decreased by Hpo overexpression. (b) Ectopic Hpo results in decrement of larval triglyceride content. Triglyceride content of third instar larvae expressing either control or Hpo transgene driven by *Dcg-Gal4* was quantified (data were expressed as mean ± SEM from three independent experiments. *p<0.05, n>6 for each genotype). (c) Ectopic Hpo expression results in small body size in adult flies. The photograph was taken from well-fed female adults expressing control or Hpo transgene under the control of *Dcg-Gal4*. (d) Ectopic Hpo reduces adult body weight. Body weights of five to seven-day-old adults expressing control or Hpo transgene were examined. Data were expressed as mean ± SEM from three independent experiment. *p<0.05 **p<0.01, n>20 for each genotype. (e) Ectopic Hpo causes decrement of triglyceride content in adult flies. Triglycerides of five to seven-day-old adults were quantified. Note that overexpression of Hpo significantly inhibits fat storage in adult male flies. (f) Ectopic Hpo leads to early death in starved flies. Five to seven-day-old male adults were deprived of food and their survival rate was plotted. Note that gain-of-function of Hpo led to more rapid death compared to the control group.

The Hpo pathway has been identified as one of the key pathways that regulate growth among different cell types, such as *Drosophila* imaginal discs [5] and intestinal stem cells [43], [44]. However, whether the Hpo pathway participates in *Drosophila* fat cell growth is unknown. In order to determine whether the Hpo pathway regulates *Drosophila* fat storage, we generated transgenic flies that express fat body-restricted Hpo under the control of the *Dcg-Gal4* driver. Since DCg1(type IV collagen) encodes gene products involved in fat cell metabolism and serves as an early terminal fat-cell differentiation maker from early stage 15 [34], the *Dcg-Gal4* driver is believed to drive gene expression in late precursor fat cells, larval and adult fat bodies [38]. Interestingly, overexpression of Hpo in fat bodies resulted in reduction of larvae size (Figure 1a). Furthermore, we observed that the Dcg-GFP (a fat reporter [37]) fluorescence signal was decreased by ectopic Hpo expression (Figure 1a). Triglyceride content, which serves as the major component of stored fat [33], was quantified to determine the level of fat storage of the larvae. As shown in Figure 1b, we noticed that the triglyceride content of larvae was dramatically reduced with overexpression of Hpo. These results were further confirmed through staining fat bodies with Oil Red, a lipophilic dye whose absorbance intensity reflects fat content. As illustrated in Figure S1a, ectopic Hpo expression resulted in a reduction of Oil Red absorbance, indicating that gain-of-function of Hpo affects fat storage during the larval stage.

As the larval fat body cells are eventually removed in adult flies after metamorphosis [45], whether Hpo regulates the size and fat storage of adult body in adult *Drosophila* is fascinating. To examine this, we crossed *UAS-Hpo* flies with *Dcg-Gal4* lines and analyzed the adult progeny. As shown in Figure 1c, overexpression of Hpo in adult fat bodies reduced the overall size of flies compared to the control group. Furthermore, we found that high level of Hpo reduced the body weight of both female and male flies (Figure 1d). Consistent with results mentioned above, triglyceride quantification to determine the fat storage level of adult flies revealed that ectopic Hpo could also reduce adult fat storage (Figure 1e), suggesting that Hpo constitutively modulates fat storage during different developmental stages. As high levels of fat storage are believed to cause insensitivity to nutrient deprivation, we then carried out a starvation assay to further dissect the role of Hpo in fat storage. Flies of approximately the same age overexpressing Hpo and control flies were transferred to grow under identical conditions and then deprived of all sources of energy from day five to day seven. As shown in Figure 1f, fat-body-specific expression of Hpo led to accelerated death compared to the control group. After 36-hour starvation, all of the flies overexpressing Hpo were dead, whereas fewer than half of the control flies had died, reflecting a low fat storage level by Hpo expression. In summary, the above results showed that fat-body-specific overexpression of Hpo inhibits fly growth by reducing fat storage, and further suggested that Hpo has an anti-obesity function in *Drosophila*.

### Inactivated Hpo increases body size via elevating fat storage

**Figure 2:**
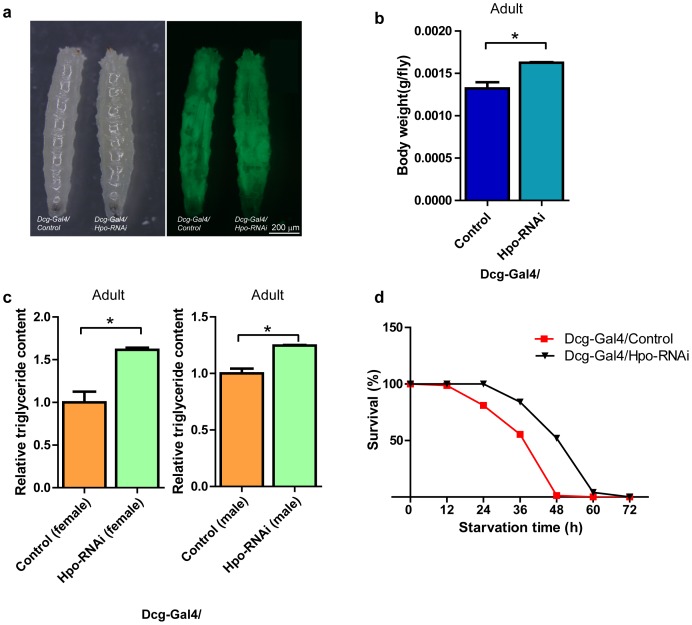
Inactivation of Hpo promotes fat storage in *Drosophila melanogaster*. (a) Compromised Hpo expression elevates larval body size and fat body reporter signal. Third instar larvae carrying a fat body GFP reporter (Dcg-GFP) expressing either control or *Hpo-RNAi* transgene driven by *Dcg-Gal4* in fat body were photographed under bright field (left) or GFP fluorescence (right) microscopy. Note that body size and GFP fluorescence were increased by knockdown of Hpo. (b–c) Inactivation of Hpo in the fat body produces obesity in adult flies. The body weight (b) and triglyceride content (c) of adult flies expressing either control or *Hpo-RNAi* transgene were quantified. Note that both body weight and fat content were increased by restricting Hpo expression. Data were illustrated as mean ± SEM from three independent experiments. *p<0.05, n>20 (b) and n>6 (c) for each genotype. (d) Knockdown of Hpo extends survival rate of starved flies. Five to seven-day-old male adults expressing control or *Hpo-RNAi* were deprived of food, and a survival curve was drawn by calculating number of the deaths every eight hours.

Since increased Hpo expression in fat bodies correlates with the reduction of fat storage, our next question was whether knockdown of Hpo in fat bodies also impacts fat storage. To address this, we expressed Hpo-RNAi under the control of *Dcg-Gal4*. As predicted, at the larval stage, loss of Hpo in fat bodies produced obese larvae compared with the control group (Figure 2a). Furthermore, expression of Hpo-RNAi was accompanied by an increase in Dcg-GFP fluorescence signal (Figure 2a), suggesting that restrictive effect of Hpo on fat storage was blocked. Loss of Hpo function also led to dramatic body weight gain in adult flies (Figure 2b). In addition, triglyceride content was upregulated when Hpo was knocked down (Figure 2c), indicating that knockdown of Hpo is capable of promoting fat storage in adult flies. A starvation assay was then carried out to evaluate whether the increase in fat storage levels observed when Hpo is inactivated is resistant to starvation. Indeed, we found that Hpo-RNAi was able to extend the survival time of flies under starvation conditions (Figure 2d). In brief, these results indicated that Hpo plays an inhibitory role in *Drosophila* fat storage and that inactivating Hpo by Hpo-RNAi has pro-obesity effects in flies.

### Activated Yki overexpression promotes fat storage and counteracts reduction of fat by Hpo expression

**Figure 3:**
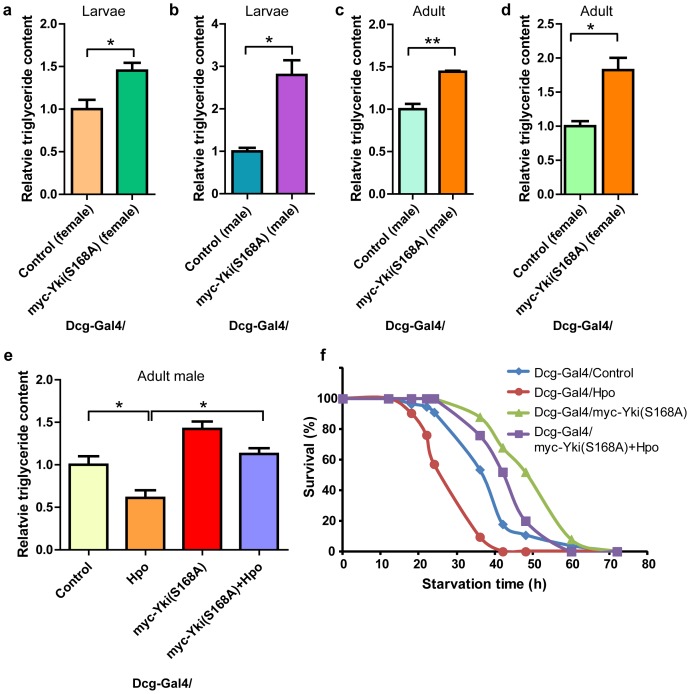
Activated Yki promotes fat storage and rescues the reduction of lipid content caused by ectopic Hpo in expression *Drosophila melanogaster*. (a–b) Gain-of-function of Yki elevates triglyceride content of larvae. Triglyceride content of male (a) and female (b) third instar larvae was examined. Note that activated Yki (Yki(S168A)) dramatically increased larval fat storage. (c–d) Gain-of-function of Yki elevates triglyceride content of adult flies. Triglyceride content of five to seven-day old male (a) and female (b) flies was quantified. Note the increase in fat content when ectopic Yki(S168A) is expressed. (e) Activating Yki suppresses the effect of Hpo on fat storage. Triglyceride content of five to seven-day old male flies expressing the indicated transgenes was analyzed. Note that overexpression of Hpo specifically in the fat body reduced fat storage, while coexpressing Yki(S168A) rescued this phenotype. Data were illustrated as mean ± SEM from three independent experiment. *p<0.05, n>6 for each genotype. (f) Ectopic Yki(S168A) promotes fly survival under starvation conditions. Adult flies expressing the indicated *Dcg-Gal4*-driven transgenes were starved, and the survival rate was examined. Note that expression of Yki(S168A) extended the survival time of flies. In addition, Yki(S168A) could reverse the pro-death effect of Hpo.

To address whether Hpo restricts fat storage through the downstream transcriptional complex of the Hpo pathway, we generated transgenic flies expressing activated Yki, a Wts un-phosphorylated mimic created by mutating Yki serine168 to alanine (hereafter referred to as Yki(S168A)), under the control of *Dcg-Gal4* to evaluate the role of Yki in fat storage. As shown in Figure 3a–b and Figure S1b, specific overexpression of Yki(S168A) in fat bodies significantly promoted fat accumulation in *Drosophila* third instar larvae. More importantly, we found that Yki(S168A) induced increment of fat storage was independent of *Drosophila* life stages since ectopic Yki(S168A) also significantly elevated fat content in adult flies (Figures 3c and 3d).

To determine the functional relationship between Hpo and Yki(S168A) with regard to fat storage, we coexpressed Hpo and Yki(S168A) in *Drosophila* fat bodies. As expected, gain-of-function of Yki(S168A) rescued the reduction of fat content induced by Hpo overexpression, although the rescue was not complete (Figure 3e). We also carried out a starvation assay to further confirm the interrelation between Hpo and Yki(S168A). As illustrated in Figure 3f, ectopic Yki(S168A) extended the survival time of flies under starvation conditions, whereas Hpo accelerated the death of flies. Interestingly, we found that early death caused by Hpo overexpression could be reverted by expressing Yki(S168A), indicating that Yki functions downstream of Hpo to control fat storage. In summary, these results affirmed the prediction that most, if not all, Hpo goes through Yki to restrict fat storage.

### Hpo controls fat cell number to affect fat storage

**Figure 4:**
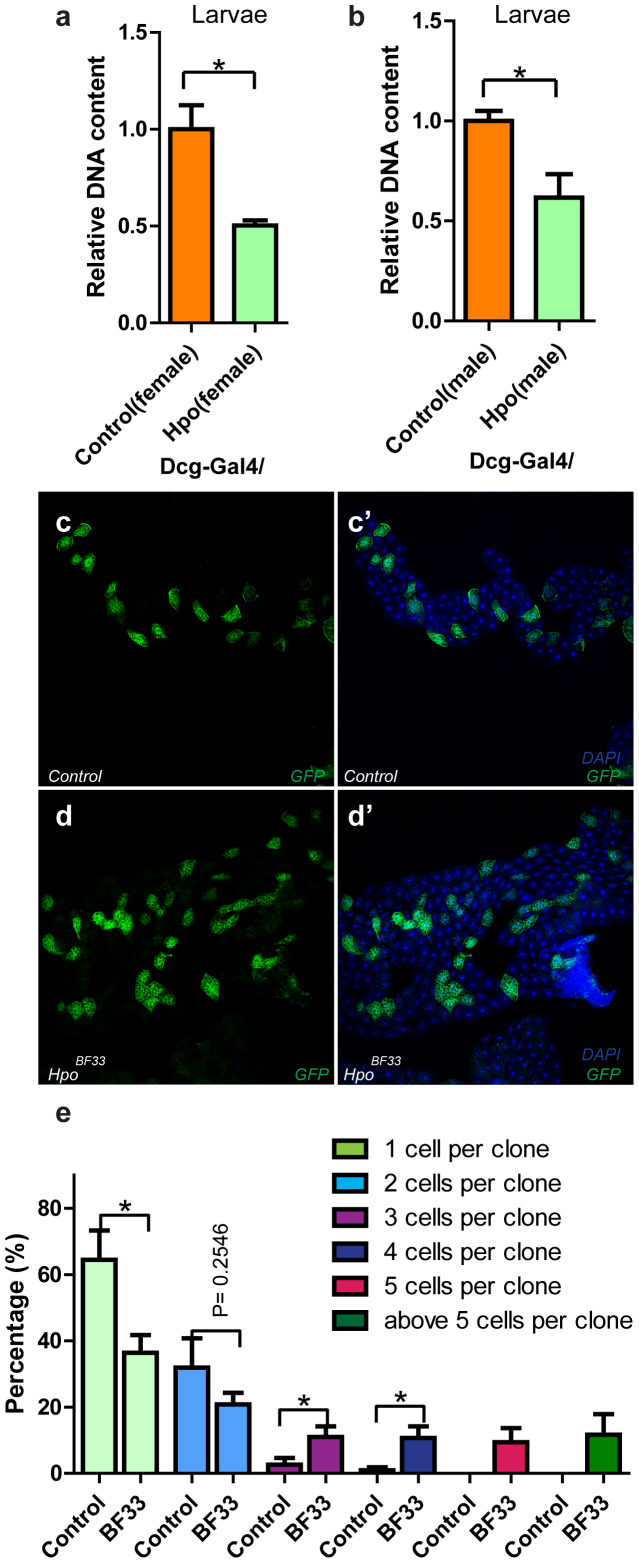
Hpo controls fat cell number to affect fat storage. (a–b) Ectopic Hpo decreases fat cell number. Hpo or control transgenes were expressed in the fat body by crossing with a fat body specific driver, *Dcg-Gal4*. To quantify fat cells, the genomic DNA of third instar larvae was extracted and quantified. Note that DNA content of both male (a) and female (b) larvae was reduced upon overexpression of Hpo. (c–d) Knockout of Hpo promotes fat cell proliferation. Mosaic analysis with a repressible cell marker (MARCM) technology was utilized to generate clones in fat body. Note that clones lacking Hpo grew larger than control clones. (e) Quantification of clone size. The number of GFP positive cells within one clone was counted. More than 20 independent microscopic fields from more than six larval fat bodies of each genotype were counted. The data were calculated as follows: the number of GFP positive cells within one clone divided by the total number of GFP positive cells in one independent microscopic field. The data were expressed as mean ± SEM, *p<0.05.

After observing the anti-obesity function of Hpo, we performed further studies to uncover the mechanism of how Hpo controls fat storage. Fat storage can be affected by fat body cell number, fat cell size, or the amount of triglyceride in each cell. As the Hpo pathway was reported to play an important role in organ size control by balancing cell proliferation and cell death, but not affecting cell size [8], we predicted that Hpo controls fat storage through affecting fat cell number rather than cell size. To verify this, we first generated Hpo flip-out clones in fat body cells. As shown in Figure S2a–a′, cells that overexpressed Hpo displayed no discernible size difference compared with neighboring cells, indicating that ectopic Hpo did not affect single fat cell growth. As it is very difficult to count fat cell number, we instead extracted the total DNA from the fat body, the amount of which is reflective of the number of fat cells. If Hpo expression reduces fat cell number, the total DNA content should decrease. Indeed, we found that expressing Hpo under the control of *Dcg-Gal4* resulted in a decrease of total DNA content of the fat body (Figures 4a and 4b), indicating the reduction of fat cell number, which may be responsible for Hpo induced fat loss. To strengthen the conclusion, mosaic analysis with a repressible cell marker (MARCM) technology was employed to generate *hpo* null clones in the fat body to evaluate the influence of Hpo knockout on controlling fat cell numbers. As expected, loss of Hpo produced larger clones compared with control clones (Figure 4c–c′, and Figure 4d–d′). In the control group, more than half of the clones consisted of one cell and failed to grow into larger clones with more than five cells (Figure 4e), indicating that fat cells undergo one to two rounds of cell division after clone formation. However, clones lacking Hpo expression entered into extra cell division cycles to generate larger clones. All together, these data suggested that Hpo regulates fat storage through controlling the number of fat cells.

Since fat storage is closely related to the amount of lipid in each cell, we next sought to figure out whether Hpo affects the fat content within individual cells. For this, *UAS-Hpo* was expressed in the fat body driven by *Dcg-Gal4*, and the fat body cells were dissected for Nile Red staining, which reflects the amounts of lipid in cells by measuring the intensity of lipid droplets. As shown in Figures S2b and S2c, gain-of-function of Hpo resulted in an indistinguishable Nile Red intensity and lipid droplets of single fat cell, suggesting that Hpo influences fat storage without affecting lipid metabolism of single fat cell. In brief, we provided evidence here that Hpo modulates fat storage by controlling fat cell number rather than by affecting fat cell size or amount of fat within individual fat cells.

## Discussion

Previous studies have shown that the Hpo pathway plays important roles in balancing cell proliferation and cell death among different cell types. Furthermore, accumulating evidence has suggested that inactivation of Hpo pathway components lead to multiple forms of cancer via abnormal cell proliferation. Though extensive studies have been done to address how Hpo pathway controls epithelial cells growth, whether Hpo pathway also affects the proliferation of non-ectoderm-derived cells is elusive. Here, we reported that Hpo affects *Drosophila* fat storage via controlling numbers of mesoderm-derived fat cells. Fat-body-restricted overexpression of Hpo results in a reduction of fat storage, while knockdown of Hpo in fat bodies promotes fat accumulation. In addition, we provided evidence that Hpo goes through a downstream executor, Yki, to modulate fat storage. Our study elucidates the anti-obesity function of Hpo, which may provide insight into the treatment of obesity.

Although a novel function of the Hpo pathway was proposed, some unsolved problems should be addressed in further investigation. Obesity is the result of imbalance between caloric intake and expenditure, and manifests itself as an increase in adipocyte size or number. As overexpression of Hpo was able to reduce the number of fat cells in *Drosophila*, substantial amount of further work, especially studies using mammalian systems may be able to make activation of Hpo into a promising strategy for combating obesity.

In the present study, we provided evidence that Hpo affects fat storage via controlling fat cell number, but how Hpo regulates fat cell number is yet unclear. As we all know that, Hpo controls organ size by balancing cell proliferation and inducing apoptotic cell death. However, it is still unknown whether reduction of fat cell number induced by ectopic Hpo expression is due to defective cell proliferation, apoptotic cell death, or other reasons. As no discernible apoptotic cell death was observed via terminal deoxynucleotidyl transferase dUTP nick end labeling (TUNEL) staining (data not shown) and it has been reported that proapoptotic genes, such as *Reaper*, *Hid*, and *Grim* are incapable of inducing apoptotic cell death in fat body during the last larval stage [46], we suspect that Hpo modulates fat cell number through affecting fat cell proliferation rather than through inducing apoptotic cell death. As larval fat cells are eventually removed by autophagy under normal physiological conditions [45], another possibility for to explain the reduction of fat cell number caused by ectopic Hpo may be due to the triggering of autophagic cell death. To examine this, Hpo overexpressed flip-out clones were generated. As shown in Figures 2d–d′″, ectopic Hpo didn't respond to Lysotracker staining, which serves as a reporter of autophagy, suggesting that the reduction of fat cell numbers resulted from Hpo is independent of autophagy. Another mystery fascinates us is that when the proliferation of fat cells was affected by Hpo pathway. We found that the differentiated fat cells lacking Hpo expression or with Yki overexpression driven by Dcg-Gal4 were incapable of re-entering into mitotic cell cycle at third instar larval stage (data not shown). Furthermore, at this stage, we observed an indistinguishable expression of Hpo pathway target genes (data not shown) between the fat cells expressing control transgene and the cells expressing Hpo or Yki transgenes. As Dcg-Gal4 could drive gene expression from late embryonic stage, we favor the model that Hpo pathway regulates the proliferation of undifferentiated fat cells. Since a technical limitation to dissect early stage fat cells, we suspect that further studies using inducible systems to drive Hpo expression may solve the mystery of how and when Hpo affects fat cell proliferation.

In conclusion, in this study we provided evidence that fat body proliferation is affected by Hpo pathway, which may indicate great potential for utilization of Hpo in anti-obesity therapeutics.

## Supporting Information

Figure S1 **Activating Hpo inhibits fat accumulation in** ***Drosophila melanogaster*** **, whereas ectopic Yki promotes fat storage.** (a) Ectopic Hpo results in decrement of larval fat content. The fat content of third instar larvae expressing either control or Hpo transgene driven by *Dcg-Gal4* was determined by Oil Red Staining. (b) Activating Yki increases fat content of larvae. The fat content of third instar larvae expressing either control or Yki(S168A) transgene driven by *Dcg-Gal4* was determined by Oil Red Staining. Data were expressed as mean ± SEM from three independent experiments. ***p<0.001, n>6 for each genotype.
(TIF)

Figure S2 **Ectopic Hpo has no effect on fat cell size or lipid storage of a single fat cell and does not induce autophagic cell death.** (a–a′) Gain-of-function of Hpo is incapable of affecting fat cell size. *Drosophila* fat body containing flip-out clones expressing *act>CD2>Ga4-*driven *UAS-Flag-Hpo* were dissected and immunostained with the indicated antibodies. Cells expressing the *UAS-Flag-Hpo* transgene were labeled by Flag tag (white arrow). Note that clones with Hpo overexpression showed no discernible difference in size from their neighboring cells (green arrow). (b–c) Overexpression of Hpo has no effect on lipid storage of a single fat cell. *Drosophila* fat body expressing *UAS-Flag-Hpo* or control transgenes were dissected and stained with Nile Red. Note that the intensity of Nile Red and the lipid droplets of single fat cell were not affected by Hpo expression. (d–d′″) Overexpression of Hpo has no effect on autophagic cell death. *Drosophila* fat body containing flip-out clones expressing *UAS-Flag-Hpo* by *act>CD2>Gal4;UAS-GFP* were dissected and immunostained with the indicated antibodies. Cells expressing *UAS-Flag-Hpo* transgene were labeled by GFP. Clones not expressing the *UAS-Flag-Hpo* transgene were indicated by white arrow. Lysotracker was used to detect autophagic cell death. Note that the Lysotracker pattern of clones with Hpo overexpression showed no discernible difference from their neighboring cells.
(TIF)
