# Supplementary figures and images for: Drosophila Ste-20 Family Protein Kinase, Hippo, Modulates Fat Cell Proliferation
Source: PLoS One. 2013 Apr 18;8(4):e61740. doi: 10.1371/journal.pone.0061740 (PMC3630116; doi:10.1371/journal.pone.0061740)

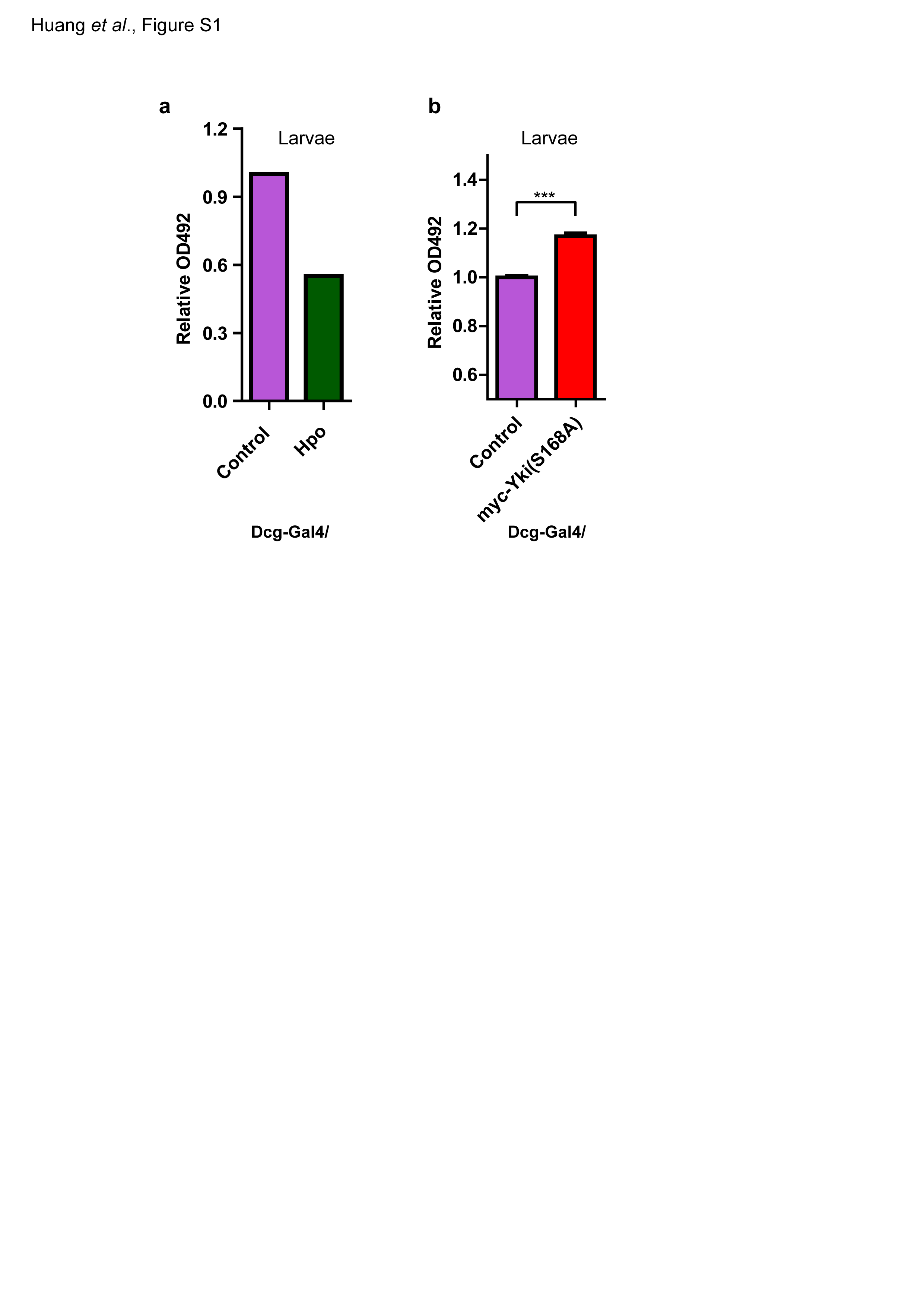

Supplement: Figure S1 — Activating Hpo inhibits fat accumulation in Drosophila melanogaster , whereas ectopic Yki promotes fat storage. (a) Ectopic Hpo results in decrement of larval fat content. The fat content of third instar larvae expressing either control or Hpo transgene driven by Dcg-Gal4 was determined by Oil Red Staining. (b) Activating Yki increases fat content of larvae. The fat content of third instar larvae expressing either control or Yki(S168A) transgene driven by Dcg-Gal4 was determined by Oil Red Staining. Data were expressed as mean ± SEM from three independent experiments. ***p<0.001, n>6 for each genotype. (TIF) [file pone.0061740.s001.tif]

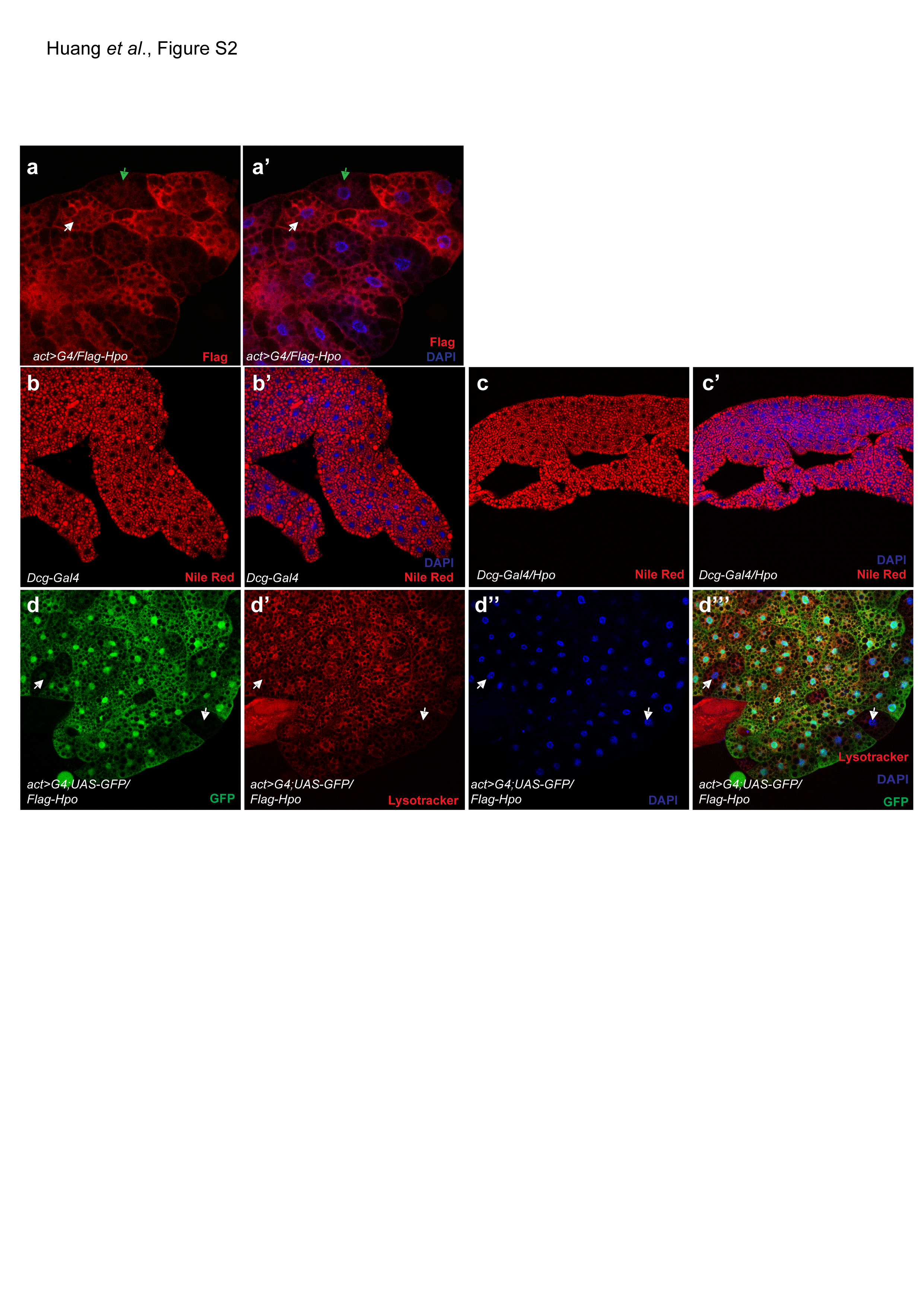

Supplement: Figure S2 — Ectopic Hpo has no effect on fat cell size or lipid storage of a single fat cell and does not induce autophagic cell death. (a–a′) Gain-of-function of Hpo is incapable of affecting fat cell size. Drosophila fat body containing flip-out clones expressing act>CD2>Ga4-driven UAS-Flag-Hpo were dissected and immunostained with the indicated antibodies. Cells expressing the UAS-Flag-Hpo transgene were labeled by Flag tag (white arrow). Note that clones with Hpo overexpression showed no discernible difference in size from their neighboring cells (green arrow). (b–c) Overexpression of Hpo has no effect on lipid storage of a single fat cell. Drosophila fat body expressing UAS-Flag-Hpo or control transgenes were dissected and stained with Nile Red. Note that the intensity of Nile Red and the lipid droplets of single fat cell were not affected by Hpo expression. (d–d′″) Overexpression of Hpo has no effect on autophagic cell death. Drosophila fat body containing flip-out clones expressing UAS-Flag-Hpo by act>CD2>Gal4;UAS-GFP were dissected and immunostained with the indicated antibodies. Cells expressing UAS-Flag-Hpo transgene were labeled by GFP. Clones not expressing the UAS-Flag-Hpo transgene were indicated by white arrow. Lysotracker was used to detect autophagic cell death. Note that the Lysotracker pattern of clones with Hpo overexpression showed no discernible difference from their neighboring cells. (TIF) [file pone.0061740.s002.tif]
